# Supplementary material for: Turbulence simultaneously stimulates small- and large-scale CO2 sequestration by chain-forming diatoms in the sea
Source: Nat Commun. 2018 Aug 3;9:3046. doi: 10.1038/s41467-018-05149-w (PMC6076325; doi:10.1038/s41467-018-05149-w)
Supplement: Supplementary file 1 — Supplementary Information [file 41467_2018_5149_MOESM1_ESM.pdf]

Supplementary information

Turbulence simultaneously stimulates small- and large-scale CO_2 sequestration by chain-forming diatoms in the sea

Bergkvist and Klawonn et al.

Supplementary Fig. 1: *Skeletonema* and *Chaetoceros* cell chains in the phytoplankton community. The scale bar represent 20 μm.

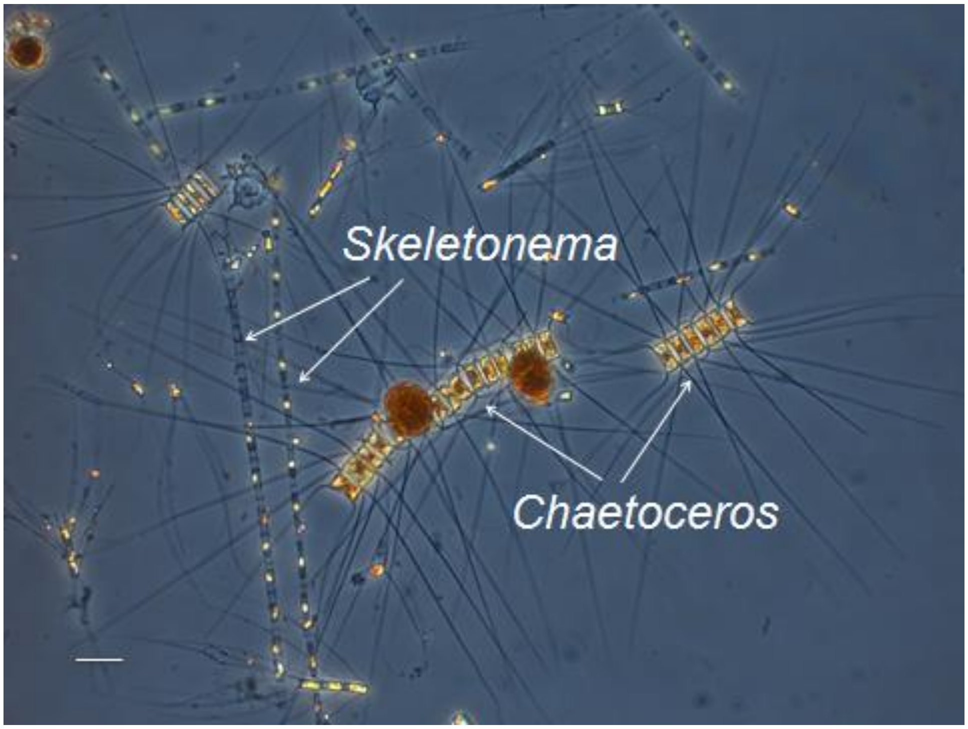

Supplementary Table1.

Cell-specific, light dependent ammonium assimilation rates. In situ ammonium assimilation rates (fmol N cell^{-1} h^{-1}) analysed by SIMS in *Skeletonema* and *Chaetoceros* during day and night. The rates represent the average value with s.d.. Number of cells analysed are indicated by n.

**Genera**	**Daytime**<br>**Cell-specific NH_4^+ assimilation rate**<br>**(fmol N cell^{-1} h^{-1})**	**Night time**<br>**Cell-specific NH_4^+ assimilation rate**<br>**(fmol N cell^{-1} h^{-1})**
Skeletonema	0.87 ± 0.52 (n= 93)	1.61 ± 0.76 (n=42)
Chaetoceros	5.6 ±3.8 (n= 138)	7.8 ±3.4 (n= 129)
